# Case Report: Intra-articular injection of tocilizumab for arthritis treatment in chronic graft-vs.-host disease

**DOI:** 10.3389/fped.2025.1515706

**Published:** 2025-07-02

**Authors:** Mingyu Xie, Qin Liu, Huiting Yuan, Yumei Chen, Xian Zou, Zhenhong Zhang, Baimao Zhong, Huasong Zeng, Haisheng Zeng

**Affiliations:** ^1^ Department of Pediatric Rheumatology and Immunology, Dongguan Children’s Hospital Affiliated to Guangdong Medical University, Dongguan, Guangdong, China; ^2^ Rare Diseases Diagnosis & Treatment Center, Dongguan Children’s Hospital Affiliated to Guangdong Medical University, Dongguan, Guangdong, China; ^3^ Accurate Diagnosis & Key Laboratory of Rare Disease in Children, Dongguan Children’s Hospital Affiliated to Guangdong Medical University, Dongguan, Guangdong, China; ^4^ Department of Pediatric Allergy, Immunology & Rheumatology, Women and Children’s Medical Center, Guangzhou Medical University, Center and South National Pediatric Medical Center, Guangzhou, China

**Keywords:** cGVHD, tocilizumab, arthritis, intra-articular injection, Case Report

## Abstract

**Background:**

Chronic graft-vs.-host disease (cGVHD) is a major complication of allogeneic hematopoietic cell transplantation. It is a leading cause of long-term morbidity, non-relapse mortality, and impaired health-related quality of life. cGVHD is a multifactorial syndrome that can manifest with articular involvement. Approximately 50% of cGVHD survivors do not respond to glucocorticoid therapy used for arthritis. Subsequently, we shall present a case of a juvenile patient with arthritis and cGVHD, who responded well to intra-articular injection of tocilizumab, after bone marrow transplantation.

**Case pressentation:**

A male adolescent with acute myeloid leukemia successfully underwent marrow stem cell transplantation. However, he developed arthritis in the elbow and knee joints and had difficulty walking more than 3 months after transplantation. He was administered anti-rejection drugs with cyclosporine, ruxolitinib, and methylprednisolone by his physician, which did not work. He was subsequently treated with intravenous tocilizumab under the supervision of his rheumatologist. Although his clinical symptoms showed remission at early stages, his knee joints were more swollen, and he could not stand after being infected with COVID-19. Both of his knee joints was injected with tocilizumab at 0, 2, 4, 6, 7, 11, and 19 weeks. Interleukin (IL)-6 levels in the peripheral blood continuously decreased. After treatment for 4 months, the patient could walk a few hundred meters with minimal exertion.

**Conclusion:**

An intra-articular injection of tocilizumab could be a viable treatment option for arthritis; however, large-scale clinical trials are warranted to confirm its efficacy.

## 1 Introduction

Allogeneic hematopoietic stem cell transfer is usually the only curative treatment for hematologic malignancies. However, it is associated with an elevated risk of developing acute or chronic graft-vs.-host disease (GVHD). Acute GVHD (aGVHD) occurs early after transplantation and primarily affects the skin, liver, and gastrointestinal tract, whereas chronic GVHD (cGVHD) occurs later after transplantation and is a complex syndrome involving multiple organ damage, including joints and fascia, lungs, liver, and skin fibrosis. cGVHD largely affects the quality of life of transplant recipients (1). The pathogenesis of cGVHD encompasses a complex multistage process, initiating with inflammatory cytokine-mediated tissue injury in the initial phase, and progressing to dysregulated tissue repair and fibrosis in later stages (2). This study reports a case of a juvenile patient with arthritis and cGVHD following bone marrow transplantation. The patient displayed a good clinical response to intra-articular injection of tocilizumab.

## 2 Case presentation

The patient was a 12-year-old Chinese adolescent boy. He was diagnosed with acute myeloid leukemia in January 2021. He successfully underwent marrow stem cell transplantation in August 2021. Unfortunately, he experienced intestinal rejection two months post-transplantation. He received anti-rejection drugs, including cyclosporine, ruxolitinib, and methylprednisolone from his physician.

He developed arthralgia in the elbow and knee joints with difficulty walking more than 3 months after transplantation. The antinuclear antibody (ANA), ANA profile, human leukocyte antigen B27 (HLA-B27), and cyclic citrullinated peptide (CCP) were negative. The levels of rheumatoid factors (RF) were 14.9 IU/ml (reference range: <14 IU/ml). Based on clinical manifestations and imaging results, we considered that he developed cGVHD with joint involvement (3). He was treated with intravenous tocilizumab under the supervision of his rheumatologist. After 1.5 years of treatment, his symptoms of diarrhea and arthralgia gradually improved.

**Figure 1:**
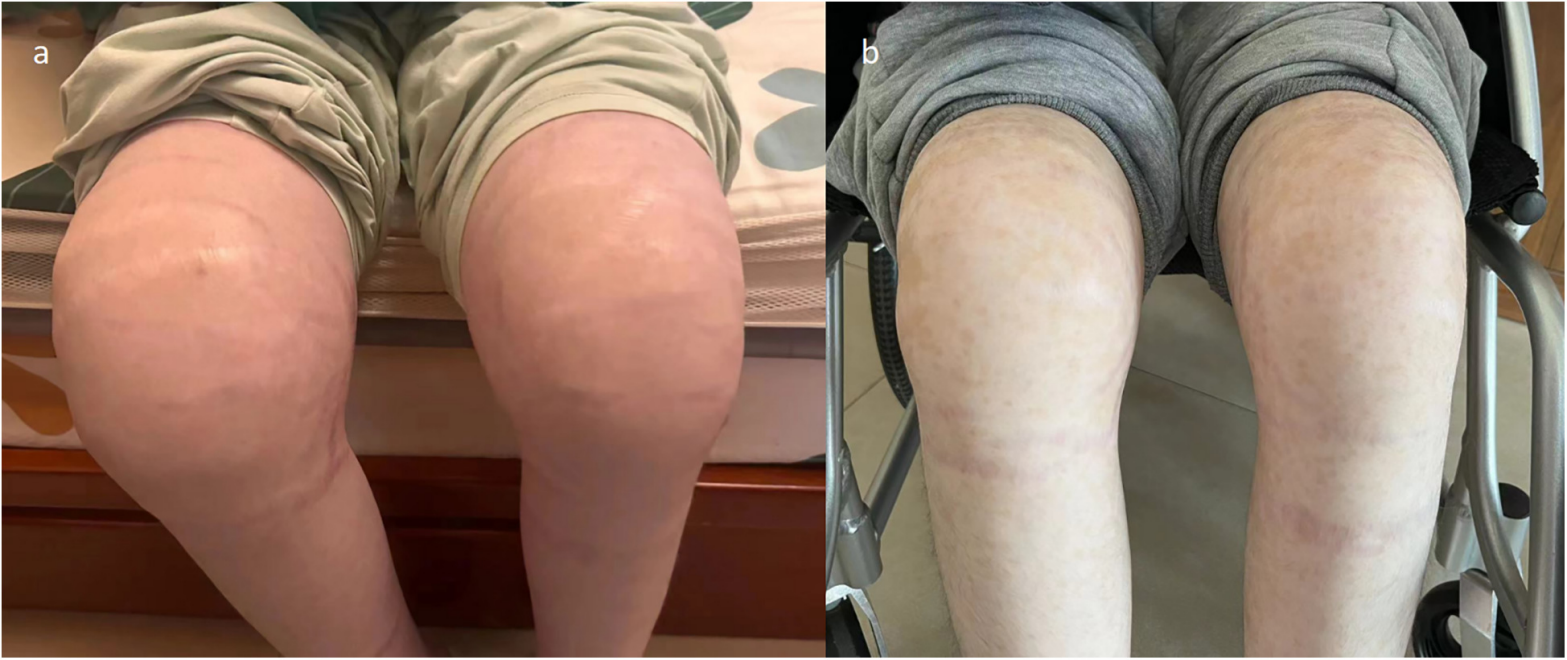
**(a)** knee joints are swollen before intra-articular injection of tocilizumab. **(b)** Knee joints swelling improved following intra-articular injection of tocilizumab.

**Figure 2:**
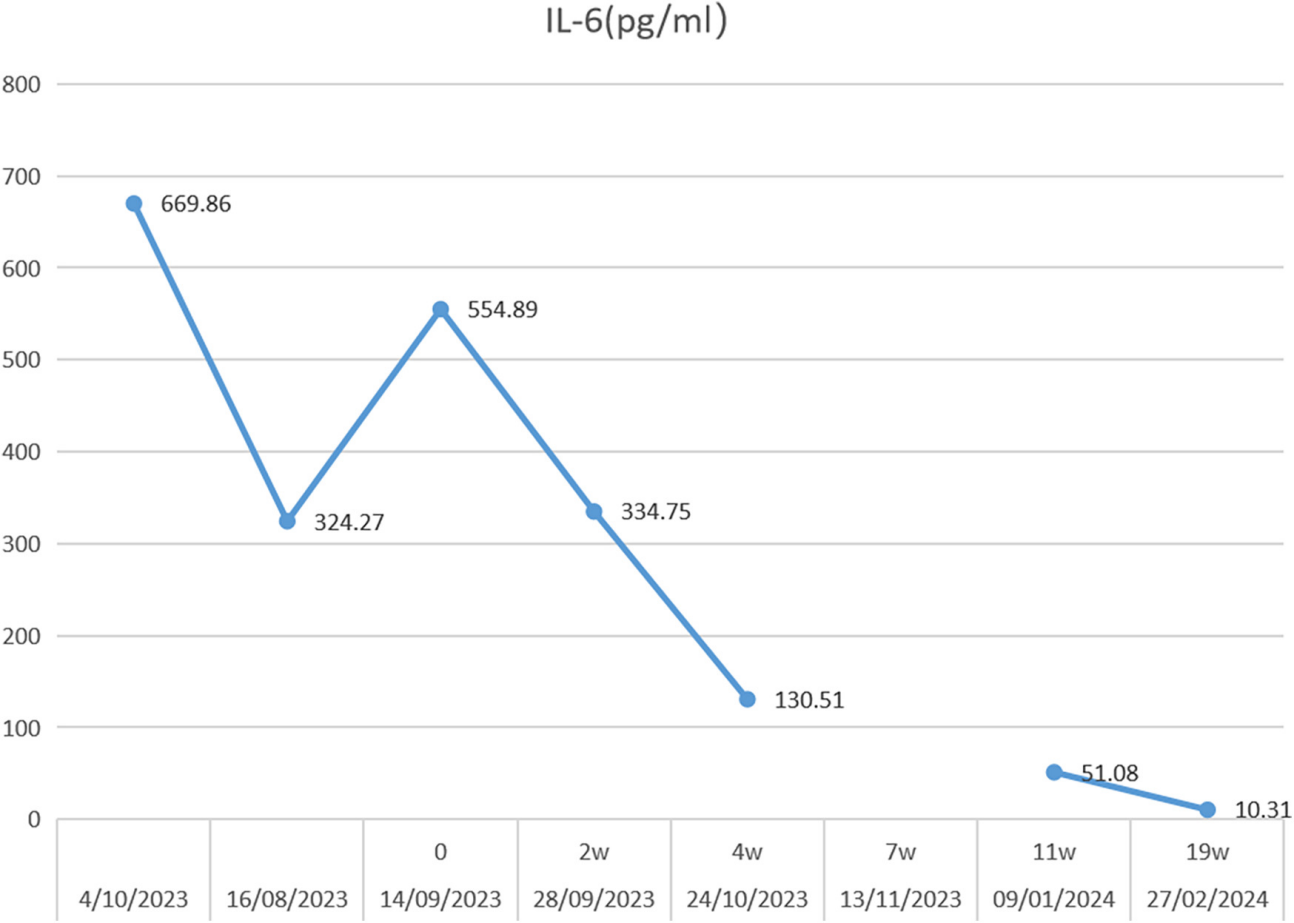
Serum IL-6 concentration trends during 19 weeks of intra-articular injection of tocilizumab.

However, he was infected with COVID-19 in January 2022, and his knee pain recurred and became worse than before, although he was still taking prednisone orally, ruxolitinib, cyclosporine, and intravenous tocilizumab. His knee joints are more swollen, and he could not stand by himself (Figure 1a and Supplementary File: Video 1). Thereafter, an ultrasound revealed abundant collection of fluid in the knee joint cavity. Magnetic resonance imaging (MRI) revealed abnormal signals in the bone marrow of the bilateral femur, patella, upper tibia, and fibula. After over 6 months of intravenous tocilizumab (dosage: 80 mg, intravenous injection, every 4 weeks) and oral ruxolitinib (dosage: 20 mg, oral, twice one day), the swelling and pain of bilateral knees did not improve. We next performed a puncture under ultrasound positioning in the joint cavity and extracted the pale-yellow fluid, revealing a significant increase in IL-6 levels in the fluid (13,940.84 pg/ml). Biochemical examination of the joint fluid demonstrated 14,045 nucleated cells, mononuclear cells accounted for 14.9%, and multiple nucleated cells accounted for 85.1%. No microbial growth was observed in synovial fluid cultures and the erythrocyte sedimentation rate (ESR) and C-reactive protein (CRP) levels were normal. We attempted intra-articular injection of tocilizumab to treat arthritis. The off-label drug treatment was recorded by the Ethics Committee of Dongguan Children's Hospital. In addition, we described the purposes and risks of intra-articular injection of tocilizumab to the patient and his parents. We both signed the risk notification for off-label drug using. The previous regimen of intravenous tocilizumab was terminated and 40 mg of tocilizumab was injected into the cavity of each knee joint under ultrasound localization. The patient was observed in the ward for 24 h after surgery. Methylprednisolone and ruxolitinib were continued for the remainder of the treatment. The same dose and treatment method were executed at 2, 4, 6, 7, 11, and 19 weeks. The level of IL-6 in peripheral blood decreased continuously (Figure 2). The patient did not experience any discomfort during intra-articular injection. After treatment for 4 months, the patient could walk a few hundred meters with minimal exertion (Supplementary File: Video 2), and the swelling of the knee joint improved (Figure 1b).

## 3 Discussion

cGVHD majorly contributes to long-term morbidity and late mortality in allogeneic hematopoietic stem cell transplant survivors (4, 5). Patients with cGVHD show involvement of multiple organs, including the skin, mouth, eyes, a small number of joints, gastrointestinal tract, lungs, liver, and genital tract (5). An epidemiological study reported that over 40% of cGVHD survivors suffer from arthropathy (6). GVHD is essentially a syndrome of autoimmune multi-organ dysfunction caused by immunological differences between the host and the donor. The pathomechanism underlying cGVHD is not completely understood. Some theories explaining the pathophysiology of cGVHD include thymic dysfunction, regulatory T cell deficiency, autoantibody production by abnormal B cells, and the formation of profibrotic lesions (7). Arthritis due to GVHD has been speculated to have a similar principle to that of rheumatoid arthritis. Glucocorticoids remain the mainstay treatment for GVHD. However, approximately 50% of patients do not respond to glucocorticoid therapy (8). No standard second-line therapy exists for steroid-nonresponsive patients. IL-6 is a pleiotropic cytokine produced by different cell types and is elevated in the serum of patients with ongoing GVHD (9–12). Tocilizumab is a humanized anti-IL-6 receptor antibody recommended for the treatment of severe rheumatoid arthritis (13). Recent studies have reported tocilizumab for the treatment of GVHD (14–16). These studies indicated that tocilizumab could be used as a remedial measure for steroid-refractory GVHD with good clinical response. However, our case study indicated that intravenous administration of tocilizumab exhibited a poor clinical effect on cGVHD accompanied by arthritis.

Intra-articular corticosteroid injections (IACIs) are largely used to treat chronic rheumatoid arthritis and osteoarthritis of the knee. IAICs aimed at joints can significantly decrease systemic adverse reactions while still achieving local anti-inflammatory effects (17). The Clinical guidelines of the American College of Rheumatology recommend IAICs as the first-line treatment for juvenile idiopathic arthritis (JIA) affecting a few joints (17). Previous studies have reported that intra-articular injection of a tumor nuclear factor (TNF) inhibitor is an effective method for treating arthromeningitis (18). We detected a significant presence of IL-6 in the synovial fluid of the patient's articular cavity. These information provide serves as a reference for envisioning the treatment of arthritis through intra-articular injection of tocilizumab. Thus, we decided to use intra-articular injection of tocilizumab to relieve knee pain.

We administered the first dose of tocilizumab in the bilateral knee cavities under ultrasound guidance. The patient exhibited no reaction following the bilateral knee joint cavity injections. Throughout the ongoing treatment, the patient's symptoms progressively improved. Serum IL-6 levels exhibited a downward trend, joint effusion was significantly reduced compared to previous instances. In a previous study, it was found that the concentration of IL-6 in the joint synovial fluid of various types of arthritis was significantly higher than that in the serum (19). This might likely be due to IL-6 being primarily secreted by synovial fibroblasts in the synovium of arthritis patients (20). IL-6 promotes the differentiation and activation of inflammatory cells, osteoclast activation, and periarticular inflammation, often working in conjunction with other pro-inflammatory cytokines. In arthrosynovitis, IL-6 significantly amplifies inflammatory responses and triggers the production of various cytokines and chemokines by acting on monocytes in the peripheral blood and synovial fluid (21). Intra-articular injection of tocilizumab may directly neutralize interleukin-6, potentially reducing local inflammation. However, additional experiments are necessary to confirm this effect.

## 4 Conclusion

Our case offers a viable treatment option for intractable arthritis. Intra-articular injection of tocilizumab could be a viable treatment for monoarticular immune arthritis, although larger clinical trials are warranted to confirm its efficacy.

## Data Availability

The original contributions presented in the study are included in the article/Supplementary Material, further inquiries can be directed to the corresponding authors.
